# Coronary CT angiography for the assessment of atherosclerotic plaque inflammation: postmortem proof of concept with histological validation

**DOI:** 10.1007/s00330-023-10169-2

**Published:** 2023-09-02

**Authors:** David C. Rotzinger, Virginie Magnin, Allard C. van der Wal, Silke Grabherr, Salah D. Qanadli, Katarzyna Michaud

**Affiliations:** 1 Division of Cardiothoracic and Vascular Imaging, Department of Diagnostic and Interventional Radiology, Lausanne University Hospital (CHUV), Rue du Bugnon 46, Lausanne, Switzerland; 2 Faculty of Biology and Medicine (FBM), University of Lausanne (UNIL), Lausanne, Switzerland; 3 University Center of Legal Medicine Lausanne-Geneva, Chemin de La Vulliette 4, Lausanne, Switzerland; 4 University Hospital of Lausanne (CHUV), Rue du Bugnon 46, 1011 Lausanne, Switzerland; 5 Department of Pathology, Amsterdam University Medical Centers (AUMC), University of Amsterdam, Amsterdam, The Netherlands; 6 Riviera-Chablais Hospital (HRC), 1847 Rennaz, Switzerland

**Keywords:** Coronary plaque, Contrast enhancement, Computed tomography angiography, Plaque characterization

## Abstract

**Objectives:**

To evaluate the diagnostic utility of multiphase postmortem CT angiography (PMCTA) to detect plaque enhancement as a surrogate marker of inflammation, using fatal coronary plaques obtained from autopsies following sudden cardiac death.

**Methods:**

In this retrospective study, we included 35 cases (12 women, 34%; median [IQR] age, 52 [11] years), with autopsy-proven coronary thrombosis, histological examination, and multiphase PMCTA. Two radiologists blinded towards histological findings assessed PMCTA for plaque enhancement of the culprit lesion in consensus. Two forensic pathologists determined the culprit lesion and assessed histological samples in consensus. Cases with concomitant vasa vasorum density increase and intraplaque and periadventital inflammation were considered positive for plaque inflammation. Finally, we correlated radiology and pathology findings.

**Results:**

All 35 cases had histological evidence of atherosclerotic plaque disruption and thrombosis; 30 (85.7%) had plaque inflammation. Plaque enhancement at multiphase PMCTA was reported in 21 (60%) and resulted in a PPV of 95.2% (77.3–99.2%) and an NPV of 28.6% (17–43.9%). Median histological ratings indicated higher intraplaque inflammation (*p* = .024) and vasa vasorum density (*p* = .032) in plaques with enhancement. We found no evidence of a difference in adventitial inflammation between CT-negative and CT-positive plaques (*p* = .211).

**Conclusions:**

Plaque enhancement was found in 2/3 of fatal atherothrombotic occlusions at coronary postmortem CT angiography. Furthermore, plaque enhancement correlated with histopathological plaque inflammation and increased vasa vasorum density. Plaque enhancement on multiphase CT angiography could potentially serve as a noninvasive marker of inflammation in high-risk populations.

**Clinical relevance statement:**

Phenotyping coronary plaque more comprehensively is one of the principal challenges cardiac imaging is facing. Translating our ex vivo findings of CT-based plaque inflammation assessment into clinical studies might help pave the way in defining high-risk plaque better.

**Key Points:**

• *Most thrombosed coronary plaques leading to fatality in our series had histological signs of inflammation.*

• *Multiphase postmortem CT angiography can provide a noninvasive interrogation of plaque inflammation through contrast enhancement.*

• *Atherosclerotic plaque enhancement at multiphase postmortem CT angiography correlated with histopathological signs of plaque inflammation and could potentially serve as an imaging biological marker of plaque vulnerability.*

## Introduction

Most myocardial infarctions (MI) are due to coronary artery disease (CAD) complicated by acute thrombotic occlusion of a coronary artery segment caused by disruption (rupture or erosion) of an atherosclerotic plaque [1]. Inflammation and neovascularization of the arterial wall occur in all stages of atherosclerosis across the initiation of lesions and progressive growth of plaques over many years [2, 3]. In addition, intraplaque inflammatory activity appears to be a critical factor in generating vulnerable “high-risk” plaques. The lesions are prone to develop ruptures or superficial erosions that evoke local thrombosis and eventually lead to the onset of ischemic syndromes [4]. Furthermore, it is now apparent that most MIs are related to plaques that lack underlying high-grade stenosis [5], suggesting that plaque vulnerability based on components such as high lipid content and inflammatory activity is a more reliable determinant of adverse cardiovascular events [6]. Plaque enhancement is a recognized marker of plaque vulnerability in cerebral and cervical atherosclerosis and is closely related to ischemic stroke [7]. Coronary artery plaque enhancement has received less attention because it is more challenging to access. Only a handful of MR studies have depicted plaque enhancement in CAD and established a link with MI [8, 9]. Nevertheless, coronary computed tomography angiography (CCTA) remains the primary noninvasive imaging modality for assessing CAD, but a surrogate marker for plaque inflammation is lacking [10].

Autopsy complemented with histology is still the gold standard for determining plaque composition, specifically plaques at risk, establishing the crucial link between CAD and death [11, 12]. Alternatively, occlusive CAD and resultant MI can be diagnosed with multiphase postmortem computed tomography angiography (PMCTA) [13]. Indeed, some histology-controlled PMCTA studies have provided encouraging results, reporting a reasonable correlation between noninvasive CT imaging and autopsy [14, 15]. While PMCTA has been applied successfully to extract data beyond lumen narrowing, studies describing plaque components in culprit lesions using PMCTA are scarce. To fill a gap in the literature, we aimed to establish the concept of coronary atherosclerotic plaque enhancement on multiphase PMCTA, with histopathology as the reference for arterial inflammation and neovascularization. PMCTA images and corresponding coronary artery specimens for this study were obtained from people who died from MI related to coronary thrombosis.

## Materials and methods

### Study population and design

**Fig. 1:**
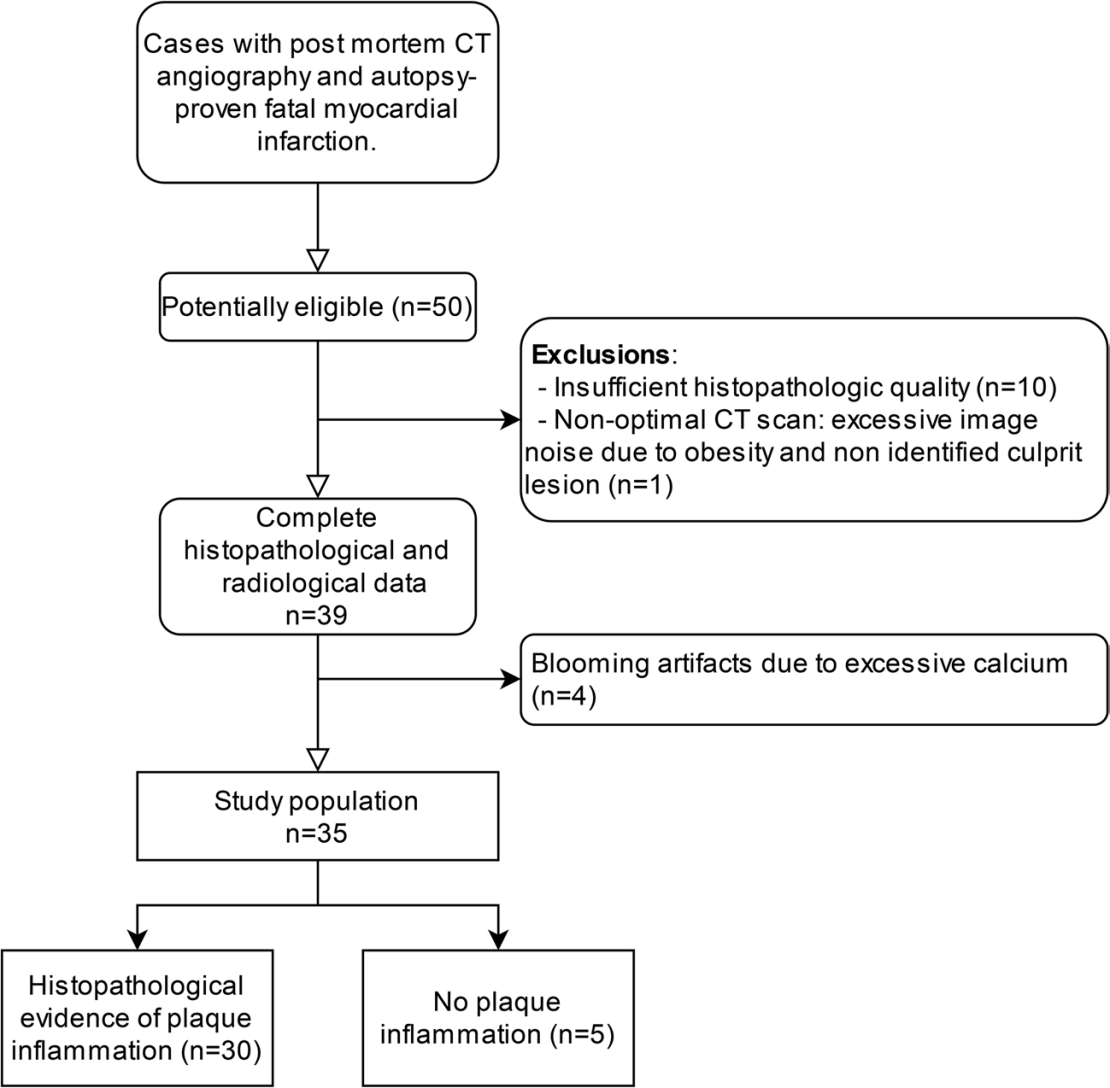
Study flow diagram

This retrospective comparative study was carried out in a single tertiary center in Lausanne, Switzerland. Figure 1 shows our study flow diagram. Our local independent Ethics Commission (CER-VD) approved this study on September 3, 2020 (protocol #2020-01530). The Lausanne University Hospital hosts a forensic department in which deceased individuals referred for autopsy systematically undergo postmortem noncontrast CT before histopathologic analysis. Additional PMCTA is performed depending on noncontrast CT findings, death circumstances, and the prosecutor’s decision. We selected consecutive cases aged 18 years or more from January 2017 to December 2020 based on MI caused by atherothrombotic CAD ascertained by medico-legal autopsy. Of 50 consecutive and potentially eligible subjects, we excluded cases with poor quality of histology due to artifacts (*n* = 10) or uninterpretable multiphase PMCTA (*n* = 1, due to excessive image noise due to obesity). Demographic data and body mass index were retrieved from autopsy data.

### Multiphase postmortem CT angiography protocol

Multiphase PMCTA was performed on a 64-row multidetector CT system (CT LightSpeed VCT, GE Healthcare) by experienced radiology technicians routinely performing postmortem CT. A dedicated cardiac noncontrast acquisition was performed, followed by several full body scans (i.e., from the head to below the hips), including arterial, venous, and dynamic phases. The following parameters were applied for each phase: mode, helical; tube potential, 120 kVp; tube current, 400 mA; beam pitch, 0.984/1; gantry revolution time, 0.8 s. As Grabherr et al [13] described, we used a standardized injection protocol involving a femoral artery and vein cannulation. This standardized protocol leads to intraluminal CT numbers that lie in the typical range of 400–500 HU used in clinical CCTA. The contrast material was an oil-based solution consisting of paraffin oil (paraffinum liquidum) with 6% oil-based contrast agent (Angiofil, Fumedica), injected through the cannulas with a dedicated power injector (Virtangio, Fumedica). We used a standard kernel and adaptive statistical iterative reconstruction (ASiR) at 60% blending, with a slice thickness of 0.625 mm, section interval of 0.4 mm, and a display field of view of 200 mm. While iterative reconstruction requires care due to image texture alteration [16], using ASiR for coronary plaque assessment was found safe without interference on plaque analysis and stenosis severity measurement, except in the 401–500 HU range where stronger ASiR levels decreased plaque volume in a previous study [17]. On the other hand, 401–500 HU represents calcified plaque while our study focuses on non-calcified components. The noncontrast, arterial, and dynamic phase images were analyzed in this study. The dynamic phase was useful when incomplete coronary artery opacification was seen in the arterial phase. On the other hand, the venous phase was not analyzed since it does not provide incremental value to detect plaque enhancement.

### Postmortem CT angiography analysis

PMCTA images were analyzed in consensus by an expert radiologist with > 10 years of experience and 5 years of forensic radiology practice (V.M.), and a fellowship-trained cardiovascular radiologist with 8 years of experience (D.C.R.). A senior cardiovascular radiologist with > 20 years of experience resolved the cases (S.D.Q.) in case of discrepancy. The observers used advanced postprocessing software (Advantage Workstation, GE Healthcare), with multiplanar and curvilinear reconstructions, and could adjust window settings in real time for optimal lumen and plaque visualization. First, the culprit lesion was identified in the coronary segment determined by histopathology. Observers could simultaneously display noncontrast, arterial, and dynamic phase images to determine plaque enhancement. We refrained from attempting CT number measurements. Image co-registering was straightforward since the corpses did not exhibit motion. Therefore, slice position could be used to synchronize different injection phases. The corresponding non-contrast image was determined manually. Plaques exhibiting visually higher attenuation in the dynamic phase were deemed positive for plaque enhancement. Furthermore, both observers evaluated the presence of a lipid core (absent; lipid core < 1 mm; or lipid core ≥ 1 mm) and spotty calcification, defined as mixed plaque with calcification measuring up to 3 mm. Mixed plaque refers to atherosclerosis containing both calcified and noncalcified components. Because calcium blooming artifacts can obscure adjacent noncalcified plaque components, cases in which the culprit lesion had gross circumferential atherosclerotic calcification were excluded (*n* = 4).

### Histopathological examination

Autopsies were performed following multiphase PMCTA on the same day, as described previously [18]. At autopsy and especially during histological examination, the main criterion to determine the culprit among multiple lesions was the presence of fresh intraluminal thrombus. Coronary thrombosis can be readily identified during autopsy and confirmed at histological examination. Once the culprit lesion was identified at autopsy, the distance from the coronary ostium to the culprit plaque was measured, and the localization was described in autopsy protocols routinely. This ostium-to-culprit distance retrieved from autopsy reports helped co-register histology with radiology employing curvilinear reconstructions of the vessel leading to the culprit lesion. Segments of coronary arteries that appeared totally occluded or at least partially (mural) thrombosed during the autopsy were collected for histological examination. Archived hematoxylin & eosin (H&E)- and trichrome-stained slides were reviewed for histopathological analysis of the occluded coronary artery segment. Histological slides were analyzed in consensus by two experts in cardiovascular pathology with > 10 years of experience (K.M., A.V.D.W.).

Intraplaque inflammation was graded on a 4-point ordinal scale as follows: 0, none; 1, small foci (0–10%); 2, moderate (10–50%); and 3, severe (> 50%). Adventitial inflammation, considered in the range of a few millimeters around the artery, was graded on a 5-point ordinal scale as follows: 1, normal (absent or scarce isolated inflammatory cells); 2, inflammatory foci occupying less than 50% of the arterial circumference, with the inflammatory zone’s thickness remaining smaller than the media’s thickness; 3, less than 50% of the arterial circumference, with the inflammatory zone’s thickness exceeding the media’s thickness; 4, inflammatory foci occupying more than 50% of the arterial circumference, with the inflammatory zone’s thickness smaller than the media’s thickness; and 5, inflammatory foci occupying more than 50% of the arterial circumference, with the inflammatory zone’s thickness exceeding the media’s thickness. Vasa vasorum density was graded on a 3-point ordinal scale as follows: 1, normal; 2, increased, less than 50% of the arterial circumference; and 3, markedly increased, more than 50% of the arterial circumference. Plaques were defined as noninflammatory when they had (a) no intraplaque inflammation or small inflammatory foci, (b) no periadventitial inflammation, and (c) normal vasa vasorum. The result of CT evaluation was classified as true/false positive or true/false negative for inflammation and vasa vasorum density increase using histopathology as the reference standard.

### Statistical analysis

In the absence of any previous CT study evaluating atherosclerotic plaque inflammation in coronary arteries, we based our statistical power calculations on gadolinium enhancement in symptomatic intracranial atherosclerotic plaques [19]. In this study, plaque inflammation was seen in 19/29 (65.5%) of culprit and 2/35 (5.7%) of nonculprit lesions. The sample size was calculated for 80% power and a 5% type-one error rate with more conservative anticipated incidences of 60% true positives and 10% false positives, under the assumption that plaque enhancement was more challenging to detect with CT than with MR. To meet these requirements, we had to include at least 26 cases. Statistical analysis was conducted using the R 3.1.3 software package (R Core Team 2015). Normality was tested for each variable group with the Kolmogorov–Smirnov test. We expressed results as the number of subjects (percentage), mean (± SD), or median (IQR) for non-normally distributed data unless otherwise specified. Categorical variables were compared using the chi-squared or Fisher exact test. Normally distributed variables were compared with the *t* test, and non-normally distributed variables by using the Mann–Whitney *U* test. Sensitivity and specificity estimates with 95% confidence intervals (CI) were computed for the PMCTA plaque enhancement sign to identify plaque inflammation against the gold standard correctly. *p* values < 0.05 were considered statistically significant.

## Results

We included 35 cases in the final analysis (median age, 52 years; range, 29–78; IQR, 11 years; 23 men). Table 1 describes the study demographics and the anatomical distribution of the culprit lesions. Histopathological analysis revealed the presence of either acute thrombosis (fibrin, platelets, erythrocyte richness) or old completely organized thrombosis in the selected coronary segment of 33/35 (94.3%) cases. Acute thrombi were due to either plaque erosion in 10/35 (28.6%) or plaque rupture in 20/35 (57%) cases. Chronic total occlusion (CTO) due to completely organized-recanalized thrombus was found in 3/35 cases (8.6%). In 2/35 (5.7%) cases, occlusive atherosclerotic plaque was observed, but the sections could not depict a thrombus. Intraplaque inflammation, presenting as infiltrates of mononuclear inflammatory cells and some neutrophils, was graded 0 in 1/35 (2.9%), 1 in 10/35 (28.6%), 2 in 15/35 (42.9%), and 3 in 9/35 (25.7%). Adventitial inflammation, presenting as mononuclear infiltrates, was graded 1 in 5/35 (14.3%), 2 in 10/35 (28.6%), 3 in 8/35 (22.9%), 4 in 3/35 (8.6%), and 5 in 9/35 (25.7%) cases. Vasa vasorum density was graded 1 in 6/35 (17.1%), 2 in 12/35 (34.3%), and 3 in 17/35 (48.6%) cases. Overall, 30/35 (85.7%) cases had histologic evidence of culprit lesion inflammation.

**Table 1:** Baseline characteristics of the study population (*n* = 35)

Demographic variables	
Women (%)	12 (34.3)
Median age, years (IQR)	52 (11)
Men	52(10)
Women	55 (12.25)
Body mass index, kg/m^2^ (± SD)	27.5 (± 6.6)
Culprit vessel (%)	
LM	1 (2.9)
LAD	11 (31.4)
LCx	2 (5.7)
RCA	21 (60)

*LM* left main, *LAD* left anterior descending, *LCx* left circumflex, *RCA* right coronary artery, *IQR* interquartile range, *SD* standard deviation

**Fig. 2:**
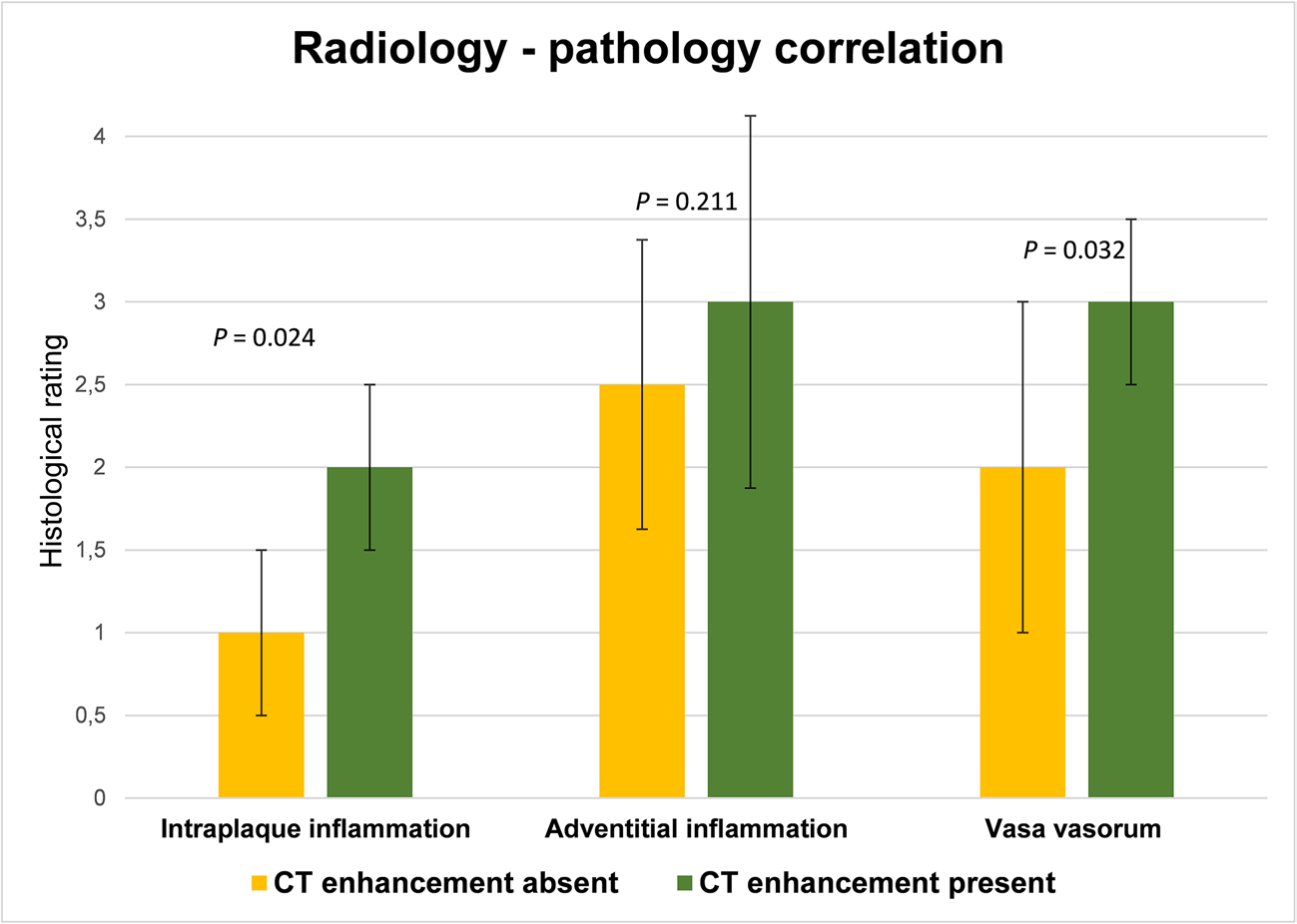
Median semi-quantitative histological grading of the extent of intraplaque inflammation, adventitial inflammation, and vasa vasorum in cases with vs. without the presence of plaque enhancement. Errors bars indicate interquartile range

Upon multiphase PMCTA analysis, plaque enhancement was detected in 21/35 (60%) cases; the remaining 14 (40%) cases constituted the control group. Most (21/35, 60%) were located in the right coronary artery (RCA), and most plaques with plaque enhancement (14/21, 66.7%) were located in the RCA. Table 2 shows the radiological-pathological relationship between multiphase PMCTA plaque enhancement and histological plaque inflammation/increased vasa vasorum. The plaque enhancement sign’s sensitivity (95% CI) was 66.7% (47.2–82.7%), the specificity was 80% (28.4–99.5%), the positive predictive value was 95.2% (77.3–99.2%), and the negative predictive value was 28.6% (17–43.9%). Cases with plaque enhancement consistently had higher scores for intraplaque inflammation, adventitial inflammation, and vasa vasorum density (Fig. 2); adventitial inflammation gradings had quite large IQRs (1.75 and 2.25, respectively) and did not reach statistical significance. Median histological ratings indicated higher levels of intraplaque inflammation and vasa vasorum density in plaques with enhancement (*p* = 0.024 and 0.032, respectively). On the other hand, we found no difference in adventitial inflammation between CT-negative and CT-positive plaques (*p* = 0.211).

**Table 2:** Presence and absence of periadventitial plaque enhancement compared with the histological gold standard

	Histology positive	Histology negative	Total
Plaque enhancement	20	1	**21**
No plaque enhancement	10	4	**14**
Total	**30**	**5**	**35**

Histological absence of plaque inflammation (“histology negative”) was defined stringently as the absence of intraplaque or perivascular inflammation, and normal vasa vasorum. Plaque enhancement was associated with histologic findings of plaque inflammation (*p* = 0.024). Please note that inflammation prevalence in this cohort was high (85.7%), but similar to in vivo studies [20]. However, the diagnostic performance only applies for scenarios with equally high prevalence

Totals are displayed in bold

**Fig. 3:**
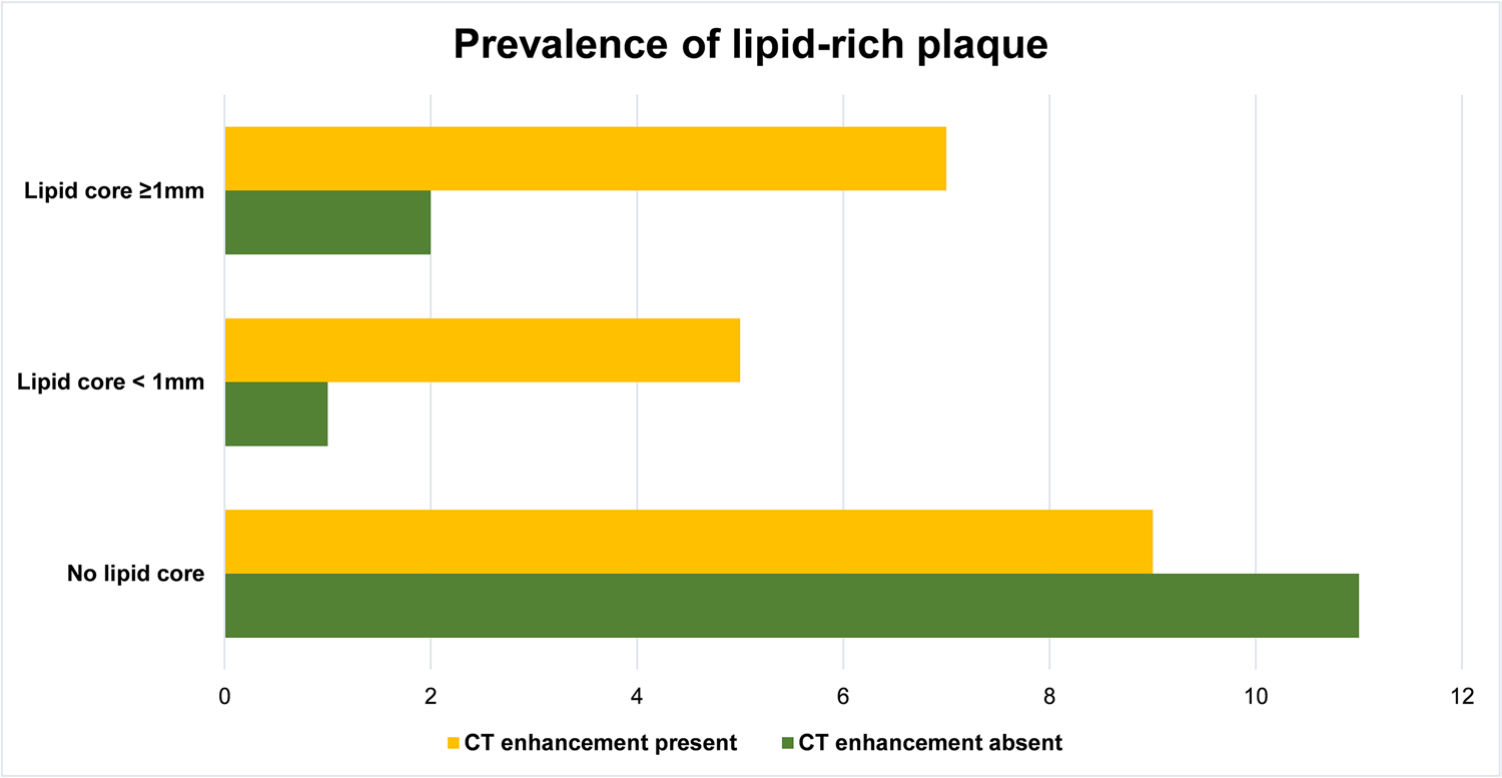
Association between lipid core and plaque enhancement on multiphase postmortem CT (number of cases). Overall (< 1 mm and ≥ 1 mm together), lipid core was more prevalent in plaques with enhancement (57.1%) than in plaques without (21.4%), *p* = 0.046. Larger lipid core size (defined as ≥ 1 mm), however, did not have a stronger association with plaque enhancement than did smaller lipid-rich plaque (< 1 mm, *p* = 0.792)

**Fig. 4:**
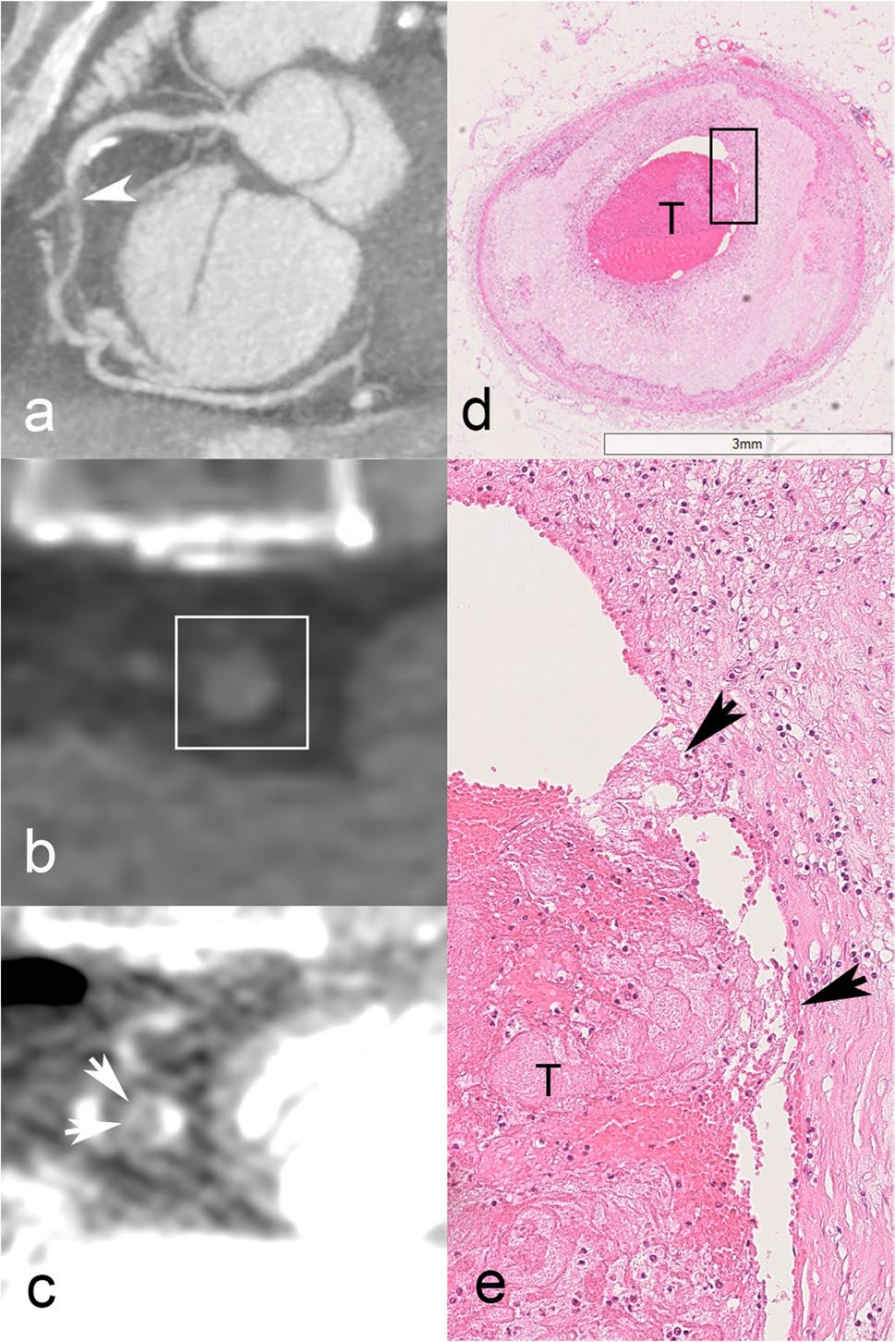
Multiphase postmortem CT angiography (PMCTA) images of a 53-year-old woman who died suddenly. Coronal reformatted maximum intensity projection image in the arterial phase depicts the right coronary artery (RCA) and high-grade stenosis in the mid-segment (**a**), corresponding to the fatal culprit lesion (white arrowhead). Cross-sectional view of the mid-RCA (**b**) at the level of the culprit lesion (white rectangle) on pre-contrast CT, showing no spontaneous density. On dynamic phase PMCTA (**c**), rim plaque enhancement (white arrows) adjacent to the plaque becomes apparent, corresponding to plaque inflammation. At histology, intraplaque inflammation was graded 3, adventitial inflammation 2, and vasa vasorum density 3. A histology section (H&E stain) of the same RCA (**d**) shows high-grade stenosis due to atherosclerotic plaque and shows acute (fresh) thrombotic occlusion (T), and underlying plaque erosion. Histology at a higher magnification of the area within the black rectangle (**e**) confirmed the presence of severe chronic intraplaque inflammation (mononuclear inflammatory cells including foamy macrophages) adjacent to eroded plaque surface (black arrows) and thrombus (T)

**Fig. 5:**
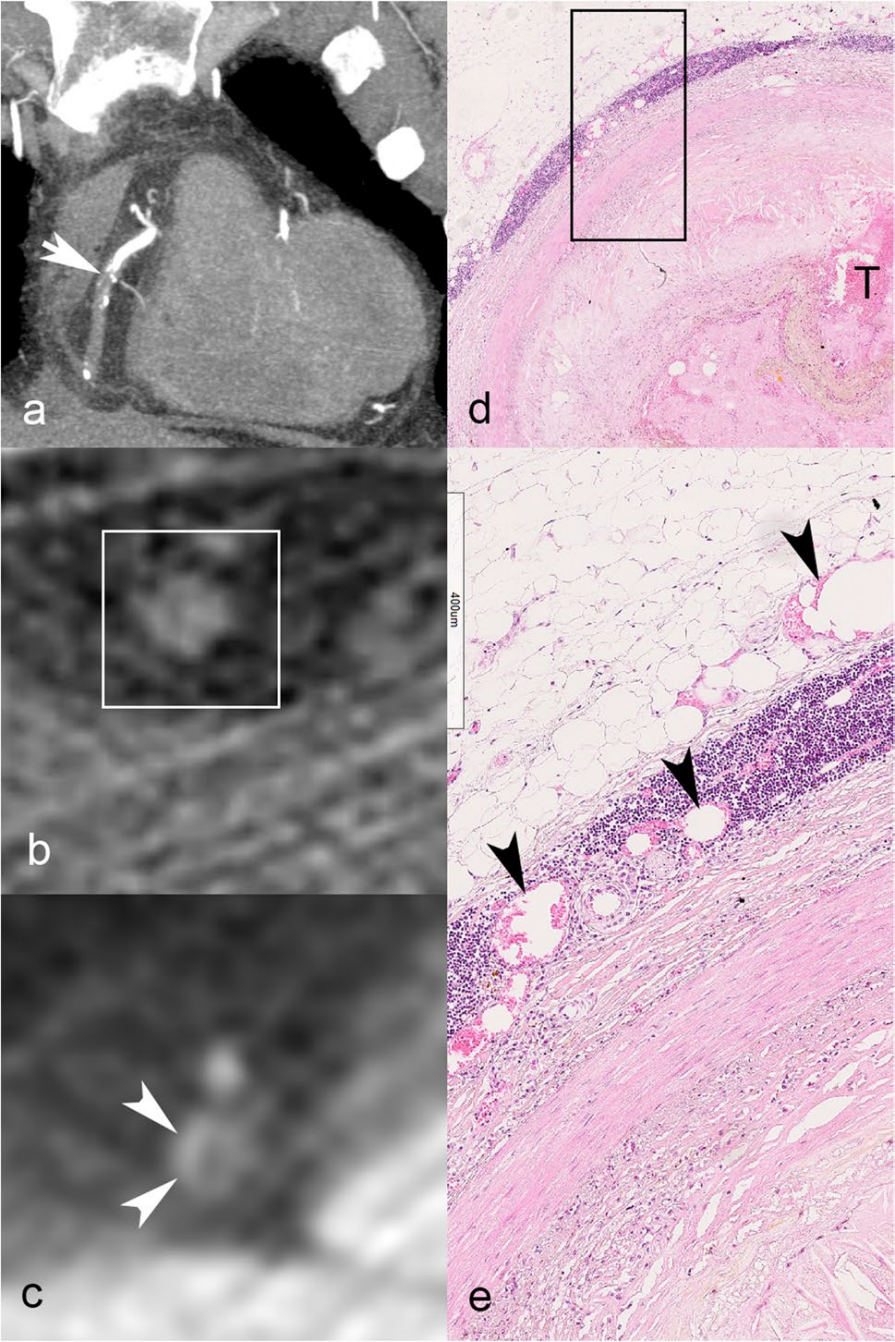
Multiphase postmortem CT angiography (PMCTA) images of a 52-year-old man who collapsed suddenly. Coronal reformatted maximum intensity projection image in the arterial phase depicts the right coronary artery (RCA) and an occlusion in the mid-segment, corresponding to the fatal culprit lesion (white arrow, **a**). Axial pre-contrast image through the RCA occlusion (white rectangle, **b**) shows no calcification, and the corresponding slice on dynamic phase PMCTA reveals contrast enhancement in the arterial wall (**c**, white arrowheads). At histology, intraplaque inflammation was graded 2, adventitial inflammation 5, and vasa vasorum density 3. **d** Histological cross section (H&E stained) through the occluded RCA segment, showing lipid-rich atherosclerotic plaque with disruption of the fibrous cap and superimposed thrombus in the lumen. The upper-left area shows epicardial adipose tissue and bandlike inflammatory infiltrates in close apposition to the arterial wall. **e** High magnification of the boxed area in **d**, showing a detail of the bandlike infiltrate composed of mononuclear inflammatory cells surrounding many (often dilated) microvessels, representing vasa vasorum (black arrowheads)

A lipid-rich core was associated with plaque enhancement: 3/14 (21.4%) cases without and 12/21 (57.1%) cases with plaque enhancement (*p* = 0.046). Figure 3 shows the lipid core analysis breakdown as absent, < 1 mm diameter, and ≥ 1 mm diameter lipid core; lipid core size was not associated with plaque enhancement (*p* = 0.792). Spotty calcification was present in roughly half of the plaques (19/35, 54.3%) and distributed similarly across plaques without (8/14, 57.1%) and with plaque enhancement (11/2, 52.4%); *p* = 1.00. A typical case of inflamed atherosclerotic plaque with superimposed thrombosis is provided in Fig. 4, with corresponding multiphase PMCTA correlation demonstrating plaque enhancement. Figure 5 shows an example of a fatal plaque with a marked increase in vasa vasorum density, which also translated to plaque enhancement on multiphase PMCTA.

## Discussion

Our results show that the majority (85.7%) of culprit plaques had histopathological signs of inflammation and increased vasa vasorum density, highlighting the critical role of inflammation and neovascularization in coronary plaque rupture and subsequent death. We report a radiological sign of plaque enhancement, established by comparing noncontrast CT with postmortem computed tomography angiography (PMCTA); this sign had a high positive predictive value (95.2%) in identifying plaque inflammation and increased vasa vasorum density correctly. However, it is critical to notice that the pre-test probability (inflammation prevalence) was also high, 85.7%, as mentioned earlier, which profoundly influences the diagnostic performance. Consequently, when applied in a population with a different disease prevalence, the latter has to be kept in mind. Nevertheless, a high prevalence of inflammation in patients dying from myocardial ischemia is not unrealistic. We know from clinical studies that the prevalence of macrophage infiltration—determined by optical coherence tomography—in culprit lesions of patients with acute myocardial infarction can be as high as 87.5% [20]. On the other hand, absent plaque enhancement showed a poor negative predictive value (28.6%) and offered no advantage in excluding histological plaque inflammation. This is likely related to the challenging nature of detecting low-grade inflammation noninvasively, and conventional CT appears to be, as yet, not suitable for this purpose. The fact that histological grading of adventitial inflammation had high IQRs may play a role, especially since many cases without plaque enhancement had at least some features of adventitial inflammation. The reason for higher IQRs is likely that adventitial inflammation was graded on a more complex 5-point scale, as opposed to 3 points for vasa vasorum density, and 4 points for intraplaque inflammation. Furthermore, as defined in current CCTA guidelines [21], low-attenuation plaque was significantly associated with plaque inflammation, unlike spotty calcification. This is in line with fresh cadaver studies that found lipid-rich coronary plaques to be associated with adventitial vasa vasorum and local inflammation in adjacent epicardial adipose tissue, suggesting an association with coronary plaque progression [11]. On the other hand, it emphasizes the caution recently stressed in young patients who might lack coronary artery calcium but have life-threatening obstructive CAD [27], and that calcium density of lesions appears to be inversely proportional to plaque vulnerability [28].

Despite CT imaging’s continuous technological progress, detecting coronary plaque inflammation noninvasively poses particular challenges due to the small size of plaques, partial volume averaging, low soft-tissue contrast, and the lack of validated surrogate markers for inflammation [22]. Next-generation CT systems, primarily photon-counting computed tomography (PCCT), might help address current challenges, in particular, thanks to dramatically improved spatial resolution and intrinsically lower noise, two mandatory determinants for challenging tasks such as vasa vasorum imaging [23, 24]. Researchers have used PCCT and micro-CT to demonstrate arterial vasa vasorum density quantification in animals [25] and explanted human hearts [26]. Our study observed nine false-negative CT cases, encouraging efforts to technically optimize the acquisition and raw data analysis to improve iodine detection. The efficacy of our PMCTA protocol to accurately image plaque enhancement and vasa vasorum density may be a matter of debate. In vivo vasa vasorum density has been measured with CT in the past, using standard clinical protocols and suggesting an association between plaque enhancement and ischemic events [27]. Data measuring the impact of infection and acquisition protocols on vasa vasorum visualization on CT is lacking. In that regard, our operation mode consisting of acquiring CT images under various artificial circulation conditions (arterial filling, venous filling, and circulating phase) offers an optimized protocol ensuring the best chance of success. This standardized protocol was validated against autopsy in a large multicenter study, is now established, and serves as a reference in medico-legal matters [28]. On the other hand, our approach’s extrapolability to in vivo scenarios is currently unknown, mainly because vasa vasorum imaging can be influenced by scan timing and our postmortem technique only approximates blood circulation. Furthermore, the use of older 64-row energy-integrating detector technology with first-generation iterative reconstruction can be limiting for imaging vasa vasorum. Future approaches might evaluate the impact of different CT systems, contrast media types, concentrations, and injection pressures or volumes on vasa vasorum imaging.

Several well-described high-risk features associated with future acute coronary syndromes can be identified noninvasively with CCTA [28, 29]. Other high-risk plaque features might indicate plaque inflammation and are still under investigation. Antonopoulos et al proposed that phenotypic changes in perivascular adipose tissue induced by vascular inflammation could be quantified using a CT fat attenuation index [29]. Other imaging modalities, such as [^18^F]-fluorodeoxyglucose positron emission tomography, have shown the potential to detect and quantify macrophage-rich atherosclerotic plaque noninvasively, likely indicating the vulnerable types of plaques at high risk of causing acute ischemic syndromes [30].

The clinical applicability of plaque enhancement in coronary arteries is in a very early stage of development and requires validation studies. Based on knowledge derived from carotid and intracranial atherosclerosis, where plaque enhancement has proved to be reliable in predicting future strokes [31] and intracranial stenosis aggravation [32], the coronary artery plaque enhancement sign might help predict acute coronary syndromes and potentially improve risk stratification. Experimental approaches, especially animal models of atherosclerotic plaque with cholesterol diet and mechanical injury of arteries, could help validate this concept in future works, for the assessment of both lipid-rich non-calcified plaque and plaque inflammation.

We acknowledge several limitations of this pilot study. First, we evaluated plaque inflammation only qualitatively. Quantitative assessment is desirable; however, performing thresholding or region-of-interest-based analysis on PMCTA is challenged by segmentation and registration issues between different acquisition phases; histogram evaluation of CT numbers in regions of interest having a thickness of roughly 0.9 mm would require a smaller voxel size. Additionally, the adventitial inflammation’s high IQRs might undermine the repeatability and generalizability of a quantitative analysis. Second, compared with clinical imaging, PMCTA has the edge due to the heart’s static state and the absence of limitations related to contrast medium or radiation. Notably, we did not use more contrast medium than for routine examinations. Third, although our datasets are complete, our sample size is still moderate. Yet, the number of centers performing PMCTA paralleled by thorough histological analysis is minimal, and a detailed radiology-pathology correlation, including inflammation analysis in fatal coronary artery plaques, has not been published. Fourth, this report is meant to focus on signs of plaque inflammation in culprit lesions. Non-culprit lesions were not collected at medicolegal autopsy, and the study does not comprehensively review all CT-based features of high-risk plaque that will be covered in an ongoing study. Finally, we used iterative reconstruction (ASiR), which can alter noise texture; future works would benefit from more advanced reconstruction algorithms, e.g. deep learning image reconstruction with stronger noise reduction capabilities and better noise texture preservation.

In conclusion, multiphase postmortem CT angiography plaque enhancement is prevalent in vulnerable coronary plaque and correlates with histopathological signs of plaque inflammation and increased vasa vasorum density. Our data suggest that plaque enhancement might be a sign of high-risk coronary artery disease.

## Abbreviations

ASiR: Adaptive statistical iterative reconstruction
CAD: Coronary artery disease
CCTA: Coronary computed tomography angiography
CTO: Chronic total occlusion
HE: Hematoxylin & eosin
MI: Myocardial infarction
PCCT: Photon counting computed tomography
PMCTA: Postmortem computed tomography angiography
RCA: Right coronary artery

## Declarations

### Guarantor

The scientific guarantor of this publication is David C. Rotzinger, MD, PhD.

### Statistics and biometry

No complex statistical methods were necessary for this paper.

### Study subjects or cohorts overlap

No study subjects or cohorts have been previously reported.

### Methodology

• retrospective

• observational

• performed at one institution
